# Polyol-Mediated Synthesis of Nitrogen-Containing Carbon-Dots from Tetracyanobenzene with Intense Red Fluorescence

**DOI:** 10.3390/nano9101470

**Published:** 2019-10-16

**Authors:** Roman Lehmacher, Claus Feldmann

**Affiliations:** Institute of Inorganic Chemistry, Karlsruhe Institute of Technology (KIT), Engesserstrasse 15, 76131 Karlsruhe, Germany; roman.lehmacher@kit.edu

**Keywords:** carbon dot, 1,2,4,5-tetracyanobenzene, polyol synthesis, nitrogen containing, red emission

## Abstract

Nitrogen-containing C-dots were prepared by heating (160 °C, 1 h) 1,2,4,5-tetracyanobenzene (TCB) in polyethylene glycol 400 (PEG400). The as-prepared monocrystalline C-dots were 2–4 nm in diameter and contained 24.4 wt. % of nitrogen. They showed intense fluorescence under excitation at 400–500 nm as well as under excitation at 600–700 nm. In addition to an excitation-wavelength-depending emission at 400 to 650 nm, the emission spectra exhibited a strong emission peaking at 715 nm, whose position was independent from the wavelength of excitation. For this deep-red emission a remarkable quantum yield of 69% was detected. The synthesis of nitrogen-containing C-dotswas completely performed in the liquid phase. Moreover, the C-dots could be directly dispersed in water. The resulting aqueous suspensions of PEG400-stabilized nitrogen-containing C-dots also showed intense red emission that was visible to the naked eye.

## 1. Introduction

Carbon dots (C-dots) have developed into an independent class of materials that is characterized by unique properties (e.g., inexpensive nature, chemical stability, adaptable surface functionalization, high biocompatibility, intense photoluminescence) and a wide portfolio of potential applications, ranging from optoelectronics or catalysis to medicine [1,2,3,4,5]. The photoluminescence of C-dots is a most remarkable feature typically characterized by broad excitation in the ultraviolet to blue spectral regime (300–500 nm) as well as by broad and intense emission in the blue to green spectral regime (450–600 nm) [1,3,4,5,6]. Another characteristic feature relates to the dependence of the emission on the wavelength of excitation. Thus, the emission is simultaneously red-shifted when shifting the excitation to higher wavelengths. In addition to the excitation-depending shift of the emission, the emission intensity decreases as the wavelength of the excitation increases [1,3,4,5,6]. Typically, C-dots show the strongest emission intensity in the blue to green spectral range, whereas the emission intensity in the yellow-red-infrared spectral regime is significantly lower. This behavior was ascribed to competitive loss processes [7,8]. Red and infrared emission, on the other hand, are important to enable devices with full-color emission (e.g., displays, lighting). Moreover, red and infrared emission are most interesting for biomedical application to guarantee deep-tissue penetration [5,6,9].

The synthesis of C-dots was most often performed by controlled thermal treatment of carbon or carbon-containing compounds. This may include high-temperature treatment of coal [10], the caramelization of carbohydrates [11], or more curiously, the thermal decomposition of, for instance, eggs [12] or oranges [13]. Well-controlled synthesis of C-dots via liquid-phase methods meanwhile is possible by controlled liquid-phase decomposition of sugar [14,15] or thermal decomposition of multivalent alcohols (so-called polyols) [16,17]. Polyols and especially polyethylene glycol (PEG) and its derivatives are also often used for surface stabilization of C-dots. In many cases, it is not even clear if polyols and PEG serve as surface-stabilizing agents or if the C-dots originate from the thermal decomposition of the polyols and PEG themselves [18]. Although reliable liquid-phase syntheses of C-dots have been established, the realization of C-dots showing intense red emission is still a challenge. Most often red emission was reported for nitrogen- or sulfur-doped C-dots, Eu^3+^-containing C-dots, or C-dots that were modified with plasmonic metal nanoparticles [6,19,20,21,22,23,24,25]. The quantum yield for red emission with 30%–35% is nevertheless comparably low in comparison to the blue and green emission of C-dots (up to 80%) [1,3,4,5,6,7,8].

Aiming at a reliable liquid-phase synthesis of C-dots that show intense red emission, we here present the polyol-mediated synthesis of C-dots with 1,2,4,5-tetracyanobenzene (TCB; also abbreviated in the literature as TCNB) as a precursor. To the best of our knowledge, TCB is used for C-dot preparation for the first time and results in intense red emission of water-dispersible C-dots.

## 2. Materials and Methods

### 2.1. Synthesis

Synthesis of C-dots with TCB: In a standard recipe, 180 mg of 1,2,4,5-tetracyanobenzene (TCB, ABCR, Karlsruhe, Germany, 98%) were dissolved in 50 mL of polyethylene glycol 400 (PEG400, Alfa Aesar, Karlsruhe, Germany) and heated in a round-bottomed flask under nitrogen atmosphere with a mantle heater to 160 °C. This temperature was maintained for 1 h. The proceeding decomposition of PEG400 and the formation of the C-dots can be followed by the naked eye and is indicated by the color of a yellow solution that slowly turns to a deep black suspension at 100–120 °C. Subsequent to the synthesis of natural cooling to room temperature, the resulting deep black suspensions can be directly diluted with water to obtain colloidally highly stable aqueous suspensions. Alternatively, the C-dots can be separated after addition of 350 mL of propan-2-ol by centrifugation (20,000 r.p.m., 10 min). Thereafter, the C-dots were purified three times by redispersion/centrifugation in/from propan-2-ol to obtain 130 mg of C-dots.

### 2.2. Analytical Tools

Transmission electron microscopy (TEM), high-angle annular dark-field scanning transmission electron microscopy (HAADF-STEM), and energy dispersive X-ray spectroscopy (EDXS) were conducted with a FEI Osiris microscope (FEI, Eindhoven, The Netherlands) at 200 kV, equipped with a Bruker Quantax system (XFlash detector, Ettlingen, Germany). TEM samples of the as-prepared C-dots were prepared by vacuum evaporation of aqueous suspensions at 120 °C on amorphous carbon (lacey-) film-coated copper grids.

X-ray powder diffraction (XRD) was performed with a Stoe STADI-P diffractometer (Stoe, Darmstadt, Germany) operating with Ge-monochromatized Cu-Kα-radiation (*λ* = 1.54178 Å) and Debye–Scherrer geometry.

Fourier-transform infrared spectra (FT-IR) were recorded on a Bruker Vertex 70 FT-IR spectrometer (Bruker, Ettlingen, Germany) using KBr pellets.

Thermogravimetry (TG) was performed with a Netzsch STA 409C instrument (Netzsch, Selb, Germany) applying *α*-Al_2_O_3_ as a crucible material and reference. The as-prepared C-dots were heated in a nitrogen atmosphere to 900–1100 °C with a rate of 5 °C/min. The resulting data were baseline corrected by subtracting the results of a measurement of an empty crucible. TG-IR coupling was realized with Bruker TGA/IR 588 equipment to the aforementioned FT-IR spectrometer.

Elemental analysis (EA): C/H/N elemental analysis was performed via thermal combustion with an Elementar Vario Microcube device (Elementar, Langenselbold, Germany) at a temperature of about 1100 °C.

UV–Vis spectroscopy: Optical spectra of the as-prepared C-dots were recorded on a UV2700 spectrometer (Shimadzu, Kyoto, Japan). Then 4–8 mg of the C-dots were mixed with 100–120 mg of dried BaSO_4_ (spectroscopic grade) and measured against dried BaSO_4_ as a reference.

Fluorescence spectroscopy (FL) and determination of quantum yield: Excitation and emission spectra were recorded with a resolution of ±1 nm using a photoluminescence spectrometer Horiba Jobin Yvon Spex Fluorolog 3 (Horiba Jobin Yvon, Bensheim, Germany), equipped with a 450 W Xenon lamp, double monochromators, Ulbricht sphere, and photomultiplier as the detector (90° angle between excitation source and detector). The determination of the absolute quantum yield (QY) was performed as suggested by Friend [23]. First of all, the diffuse reflection of the sample was determined under excitation. Thereafter, the emission was measured for the respective excitation wavelength. Integration over the reflected and emitted photons in wavelength range of 390–720 nm by use of an Ulbricht sphere allows calculating the absolute quantum yield. Standard corrections were used for the spectral power of the excitation source, the reflection behavior of the Ulbricht sphere, and the sensitivity of the detector. The QY was obtained for suspensions of the as-prepared C-dots in PEG400 that were adjusted to an absorbance of 0.1. The sample holder for determining the absolute quantum yield of suspensions in an Ulbricht sphere was constructed according to Friend [26].

Fluorescence lifetimes were obtained as process decay times with the above described Horiba Jobin Yvon Spex Fluorolog 3 spectrometer. The samples were prepared in quartz glass cuvettes under an inert gas atmosphere. The decay times were recorded by time-correlated single-photon counting (TCSPC) with pulsed laser diodes having their emission maximum at 424 and 633 nm. The time resolution was ≥0.1 nanoseconds. The fluorescence emission was collected at right angles to the excitation source, and the emission wavelength was selected with a monochromator and detected by a photomultiplier as a detector. The resulting intensity decays were calculated through tail fit analysis. The quality of the fits was evidenced by low *χ*^2^ values (*χ*^2^ < 1.4).

Daylight illumination: An Osram Xenophot HLX 64634 (3300 K) halogen bulb was used for daylight illumination of the C-dots. To select a certain color of light (green, yellow, red light), color filters were inserted between the lamp and light fiber. To differentiate scattered light and emitted light, moreover, photographs were taken through colored glass plates (i.e., if excitation of C-dots was performed with green light, the photographs were taken through a red glass plate to observe emitted red light only).

## 3. Results and Discussion

### 3.1. Polyol Synthesis of Carbon Dots (C-Dots)

In previous studies, we could already show the direct formation of C-dots via liquid-phase synthesis by partial decomposition of polyols [16,18,27]. To this concern, polyols such as glycerol (GLY), diethylene glycol (DEG), or polyethylene glycol (PEG400) were heated under inert conditions. The influence of the temperature (150–230 °C), the type of heating (conventional resistance heating, microwave heating), and the duration of heating (1–6 h) were examined [16]. Moreover, the influence of metal halides (e.g., ZnCl_2_, MgCl_2_) as Lewis acids and oxygen scavengers was validated. This polyol-mediated synthesis results in C-dots with a diameter of 3–5 nm at narrow size distribution, which show the expected broad-band excitation and emission at 300–500 nm and 450–700 nm, respectively [18]. Here, it needs to be noticed that the optimal temperature for C-dot formation from thermal decomposition of PEG400 is at 220 °C [16,18,27]. The highest quantum yields of polyol-made C-dots were observed with 45%–50% for blue-light emission. After the addition of TbCl_3_ or EuCl_3_, we could furthermore realize Tb^3+^- and Eu^3+^-containing C-dots [16,27]. After C-dot excitation, efficient energy transfer from the C-dot to the rare-earth metal was observed and results in the characteristic green and red line-type emission of Tb^3+^ and Eu^3+^. Here, quantum yields of 85% for Tb^3+^-modified C-dots and of 75% for Eu^3+^-modified C-dots were obtained [16,27]. These data still belong to the highest quantum yields reported for C-dots. Intense line-type emission of Tb^3+^-/Eu^3+^-modified C-dots, however, was only possible in absence of water. If H_2_O molecules coordinate to the rare earth cations, the emission intensity and quantum yield decrease dramatically, since relaxation takes place via O–H oscillations, which are considerably faster in comparison to the quantum-mechanically permitted *f–f* transitions [28].

**Figure 1:**
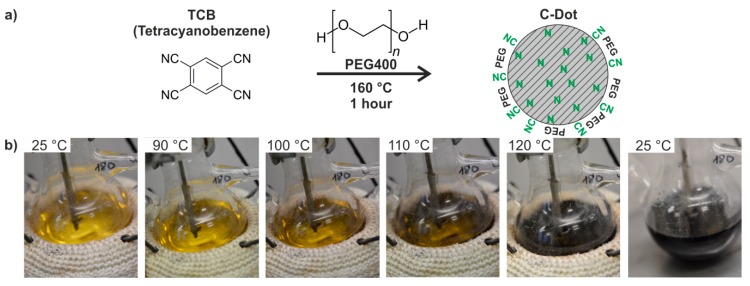
Polyol-mediated synthesis of C-dots with 1,2,4,5-tetracyanobenzene (TCB) in polyethylene glycol 400 (PEG400): (**a**) scheme of the synthesis of nitrogen-containing C-dots with PEG surface functionalization; (**b**) course of the reaction and color change at 100 to 120 °C while heating a solution of TCB in PEG400 from room temperature to 160 °C as well as dark black C-dot suspension after cooling to room temperature.

To address the challenge of C-dots that show intense red emission in aqueous suspension, we have reassumed the polyol-mediated synthesis. According to an optimized recipe, 1,2,4,5-tetracyanobenzene (TCB) was heated in PEG400 under nitrogen atmosphere for 1 h to 160 °C to obtain C-dots via thermal decomposition of TCB. PEG400 was selected as a solvent and surface-active stabilizer due to the fact that it remains in the liquid state at room temperature, whereas higher-weight PEG derivatives (≥ 500 g/mol) are extremely viscous if not solid. Furthermore, it needs to be noticed that the applied 1,2,4,5-tetracyanobenzene is also often abbreviated as TCNB in the literature. In order to avoid confusion with 1,2,4,5-tetrachloro-3-nitrobenzene, we use the abbreviation TCB throughout. TCB is well known for its photoluminescence and shows excitation at 300–400 nm and broad-band emission at 500–700 nm but only in charge-transfer crystals (e.g., with naphthalene [29]). Efficient emission of TCB is only observed for dissolved molecules (e.g., in dichloromethane [30,31]). In the solid state the emission is more or less completely quenched due to concentration quenching. Based on this situation, our intention was to examine the option of preparing nitrogen-containing C-dots based on the thermal treatment of TCB in PEG400 (Figure 1a). Accordingly, TCB was dissolved in PEG400 and simultaneously heated with the polyol at 160 °C for 1 h. The proceeding formation of the C-dots can be directly followed by the naked eye (Figure 1b). Thus, in a temperature range of 100 to 120 °C, the yellow solution of TCB in PEG400 slowly changed to a deep black C-dot suspension. Subsequent heating at 160 °C turned out as optimal to complete the synthesis and to crystallize the C-dots. Thereafter, the deep black suspension was left for natural cooling to room temperature.

Subsequent to the polyol-mediated synthesis, the as-prepared C-dots can be directly diluted with water to obtain colloidally highly stable aqueous suspensions. Alternatively, collection of the C-dots via centrifugation is possible after reduction of the high viscosity of the PEG400 suspension, for instance, by addition of propan-2-ol. Subsequently, the as-prepared C-dots can be purified by redispersion/centrifugation in/from propan-2-ol, which was typically performed three times. The deep black C-dots were obtained with about 130 mg per 50 mL of PEG400.

### 3.2. Synthesis and Characterization of C-Dots

**Figure 2:**
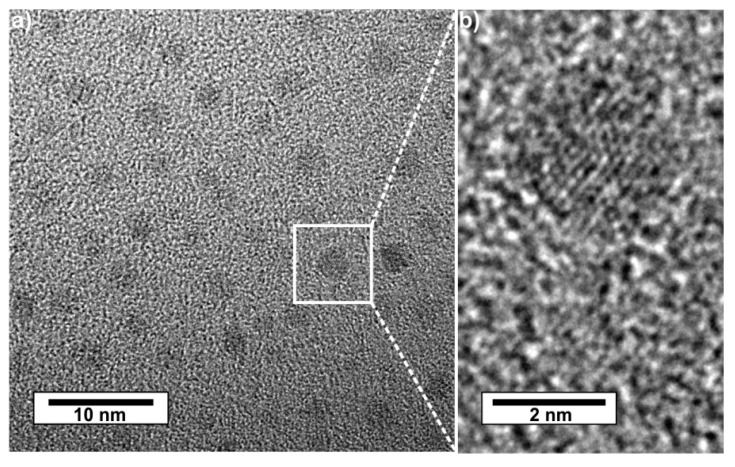
Size and size distribution of the as-prepared C-dots: (**a**) overview transmission electron microscopy (TEM) image; (**b**) high-resolution (HR)TEM image with lattice fringes.

Subsequent to polyol-mediated synthesis, separation, and purification, the presence of C-dots and their size were examined by transmission electron microscopy (TEM). Accordingly, C-dots with a spherical shape, narrow size distribution, and diameters of 2–4 nm were obtained (Figure 2). High-resolution (HR)TEM images clearly show lattice fringes and indicate the crystallinity of the as-prepared C-dots (Figure 2b). The observed lattice distance of 1.91 Å relates to graphite (*d*_101_ with 2.03 Å, [32,33]) but definitely shows a certain reduction. Such reduced lattice fringe distance was already observed for nitrogen-containing C-dots and is ascribed to the higher polarity of heteroatom-containing graphite layers in comparison to pure graphite layers [6,20,21,22,23,24,25]. In contrast, C-dots prepared via the polyol synthesis with similar conditions but in absence of TCB exhibited a larger lattice fringe distance of 2.05 Å [16,18]. In regard to the determination of the particle size, it needs to be noticed that dynamic light scattering (DLS) could not be performed since the fluorescence of the as-prepared C-dots after excitation with the DLS-internal laser (*λ_Emission_*: 633 nm) deteriorated the data evaluation.

**Figure 3:**
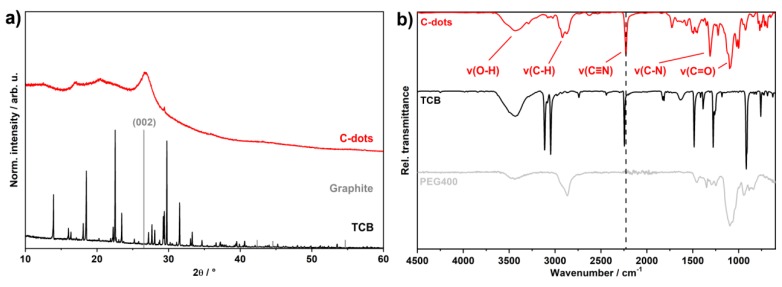
Chemical composition of the as-prepared C-dots: (**a**) X-ray diffraction pattern with pure TCB (starting material) and graphite (ICDD-No. 00-056-0159) as references; (**b**) Fourier-transform infrared spectra with pure TCB and pure PEG400 as references.

X-ray diffraction patterns (XRD) of the as-prepared C-dot powders are characterized by a broad background due to non-specific scattering. Only the most intense (002)-Bragg peak of graphite is visible at 26.6° of 2-theta (Figure 3a). The broadening and low intensity of this Bragg peak are in accordance with the small size of the C-dots. Moreover, Bragg peaks related to TCB as the starting material do not occur. Fourier-transform infrared (FT-IR) spectra show the as-prepared C-dots in comparison to pure TCB and PEG400 (Figure 3b). Due to the synthesis in PEG400, the surface of the as-prepared C-dots is inherently coated by the polyol. Thus, FT-IR spectra show the characteristic vibrations of PEG400 with *ν*(O–H) (3600–3100 cm^–1^), *ν*(C–H) (3000–2800 cm^–1^), *ν*(C=O) (1100 cm^–1^), and the fingerprint with weak *δ*(C–H)/*δ*(C–C) vibrations between 1400 and 750 cm^–1^. These vibrations are in agreement with pure PEG400 as a reference (Figure 3b). Additional vibrations at 1750–1600 cm^–1^ point to aromatic C=C bonds and indicate the formation of graphite-type C-dots [32,33,34]. Finally, the characteristic vibrations at 2228 cm^–1^ (*ν*(C≡N)) and 1315 cm^–1^ (*ν*(C–N)) validate the presence of the remaining cyano groups and point to the nitrogen-containing carbon network (Figure 3b). Interestingly, *ν*(C≡N) shifts by about 15 cm^–1^ to lower wavenumbers in comparison to pure TCB (*ν*(C≡N): 2245 cm^–1^), which can be attributed to a different chemical environment in the C-dots.

**Figure 4:**
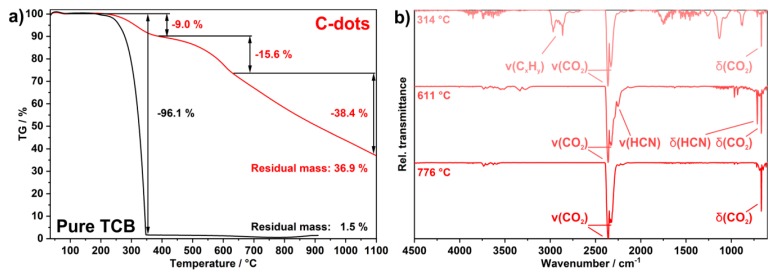
Thermal decomposition of the as-prepared C-dots: (**a**) thermogravimetry (nitrogen atmosphere, 27.7 mg of sample) with pure TCB as a reference (nitrogen atmosphere, 29.6 mg of sample); (**b**) Fourier-transform infrared spectra of the gaseous decomposition products of the thermogravimetric analysis at three selected temperatures.

The thermal behavior of the as-prepared C-dots was examined by thermogravimetry (TG) with coupled FT-IR spectroscopy to detect gaseous decomposition products (Figure 4). Upon heating to 1100 °C in a nitrogen atmosphere, three-step decomposition was observed. The first step (210–390 °C, 9.0 wt. %) can be attributed to the decomposition of surface-adhered PEG400 (decomposition and evaporation of pure PEG400 at 250–310 °C, [16,18]) (Figure 4a). FT-IR spectra monitored at 314 °C, clearly shows broad C–H- (*ν*(C–H): 3000–2800 cm^–1^) and C–O-related (*ν*(C–O): 1200–1050 cm^–1^) vibrations as well as sharp vibrations of CO_2_ (*ν*(C=O): 2360, 2330 cm^–1^, *δ*(O=C=O): 670 cm^–1^) (Figure 4b). Taking the size of the as-prepared C-dots into account (2–4 nm), a PEG400 coating with an amount of 5–10 wt. % is to be expected. The second decomposition step (400–610 °C, 15.6 wt. %) can be assigned to the release of hydrocyanic acid (HCN), which is clearly visible in FT-IR spectra recorded at 611 °C (Figure 4b). Thus, *ν*(C≡N) at 2250 cm^–1^ and *δ*(H–C≡N) at 710 cm^–1^ evidence the loss of HCN. The final continuous decomposition (>650 °C) only shows CO_2_ as a gaseous decomposition product (Figure 4b), which can be ascribed to traces of oxygen in the gas flow. The thermal residue at 1100 °C, according to XRD, still shows a broad (002)-Bragg peak of bulk graphite at 26.6° of 2-theta, which is similar to XRD patterns of the as-prepared C-dots (Figure 3a). Finally, it needs to be noticed that the thermal decomposition of the nitrogen-containing C-dots differentiates significantly from pure TCB, which evaporates completely between 250 and 350 °C (Figure 3a).

To determine the TCB load of the C-dots elemental analysis (EA) was performed, resulting in C/H/N contents of 60.5 wt. % C, 2.5 wt. % H, and 24.4 wt. % N (remaining 12.6 wt. % O). Hence, the nitrogen content was indeed considerably high. With TCB being the only nitrogen source and PEG400 being the oxygen source, the relative contributions of TCB and PEG400 to the composition of the as-prepared C-dots can be estimated from the observed C:N and C:O ratios. Accordingly, a TCB:PEG400 ratio of 6.5:1 was deduced. Based on this ratio, the calculated C/H/N/O contents result in 60.5 wt. % C, 1.5 wt. % H, 24.4 wt. % N, and 11.1 wt. % O, which is in good agreement with the experimental data. Accordingly, the yield of nitrogen-containing C-dots related to the introduced amount of TCB is 79%.

### 3.3. Fluorescence of As-Prepared C-Dots

**Figure 5:**
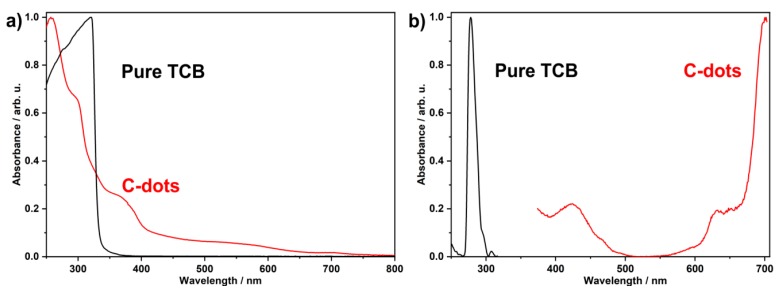
Absorption of the as-prepared C-dots: (**a**) UV–Vis spectra and (**b**) excitation spectra (suspension in PEG400, monitored at *λ_em_* = 715 nm, excitation of the C-dots could not be measured below 370 nm due to *λ_em_*/*2* peak at 357 nm). Pure TCB is shown as a reference (solution in chloroform, monitored at *λ_em_* = 332 nm).

The optical properties of the as-prepared C-dots were studied by UV–Vis as well as by fluorescence spectroscopy. UV–Vis spectra of suspensions in PEG400 indicate a strong absorption below 400 nm (Figure 5a). Distinct absorption maxima are observed at 257, 290, and 360 nm, which are clearly different from the absorption of pure TCB (Figure 5a), which only shows a strong absorption below 340 nm. These findings are confirmed by excitation spectra, which also point to the difference between the as-prepared C-dots and pure TCB (Figure 5b). In addition to the typical excitation of C-dots in the UV-to-blue spectral regime (< 500 nm), excitation spectra of the as-prepared nitrogen-containing C-dots most interestingly also show a strong absorption at 600–700 nm (Figure 5b).

**Figure 6:**
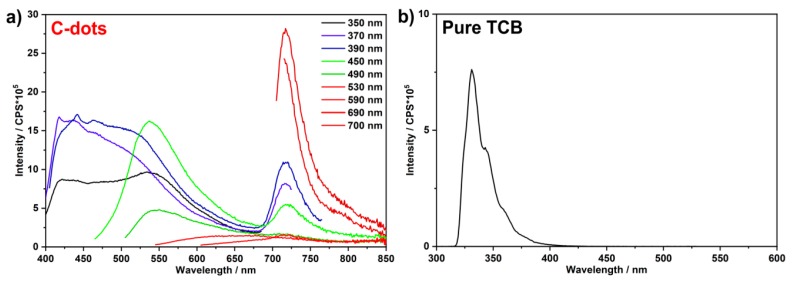
Emission of as-prepared C-dots: (**a**) emission spectra (suspension in PEG400) at different wavelengths of excitation; (**b**) emission spectrum of pure TCB (solution in chloroform, excited at *λ_exc_* = 272 nm).

**Figure 7:**
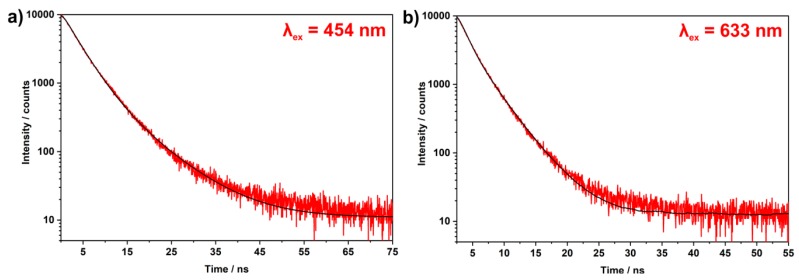
Fluorescence lifetimes of the as-prepared C-dots for the deep-red emission at 715 nm for (**a**) excitation at 454 nm and (**b**) excitation at 633 nm.

Emission spectra of the as-prepared C-dots indicate continuous emission between 400 and 850 nm with a broad maximum at 400–600 nm and a narrow maximum at 715 nm (Figure 6a). The emission at 400 to 600 nm clearly depends on the wavelength of excitation (Figure 6a). In contrast, the emission maximum at 715 nm is stationary and its wavelength position not influenced by the wavelength of excitation. The emission intensity of this deep-red emission is comparably high if excited via the C-dots at 400–500 nm with large Stokes shift (Figure 6a). The deep-red emission is comparably low upon excitation between 500 and 600 nm since the absorption of the C-dots is low in this wavelength regime. However, the deep-red emission is even by a factor of 2–3 stronger if excited above 600 nm with small Stokes shift. For long-wavelength excitation (690 nm) with subsequent deep-red emission (715 nm), moreover, a remarkably high quantum yield of 69% was determined. Excitation at 424 nm results in a slightly lower quantum yield of 50% for the deep-red emission (715 nm). This finding is to be expected due to the larger Stokes shift between excitation and emission. Lifetime measurements for the emission at 715 nm for both types of excitation (*λ_exc_* = 454 nm and *λ_exc_* = 633 nm) result in a decay of 1.4 to 4.3 ns (*τ*_1_ = 1.8, *τ*_2_ = 4.4 ns for *λ_exc_* = 454 nm; *τ*_1_ = 1.4, *τ*_2_ = 3.6 ns for *λ_exc_* = 633 nm) (Figure 7). These values are in good agreement with previously reported lifetime data of C-dots (*τ* ~ 1–10 ns) [35,36,37]. Finally, it needs to be noticed that the emission of C-dots made from TCB is significantly different from the emission of pure TCB (Figure 6b). Thus, pure TCB only exhibits an emission peak at 320–370 nm, which is independent from the wavelength of excitation. Moreover, no emission occurs above 400 nm.

**Figure 8:**
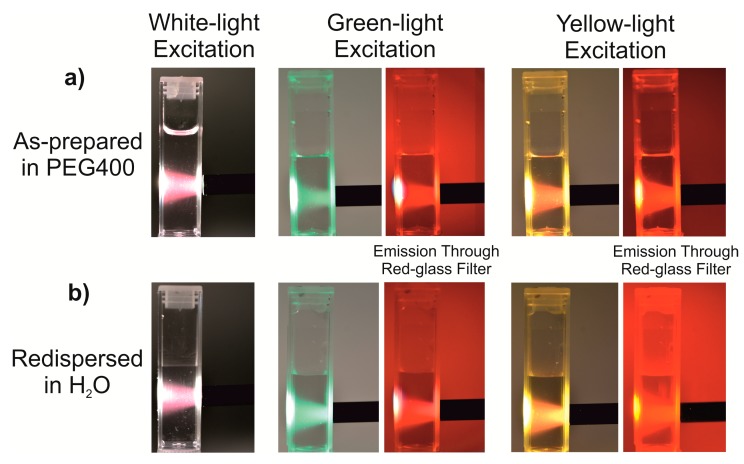
Photos showing the red emission of the as-prepared C-dots in (**a**) PEG400 and (**b**) H_2_O upon excitation with daylight, blue-green light, and yellow light. All photos were taken without a filter as well as with a red filter to prove red-light emission.

The deep red emission at 715 nm of the TCB-made C-dots can become highly interesting for biomedical application, including histology and imaging [5,6,9]. This includes both types of excitations: at the low-wavelength excitation at 400–500 nm with a large Stokes shift as well as the long-wavelength excitation at 600–700 nm with a small Stokes shift to emission (Figure 6a). Most interestingly, the deep-red emission does not only occur for the as-prepared suspensions in PEG400 (Figure 8a) but also after dispersion in water (Figure 8b). If excited with daylight, blue-green light or yellow light, the red emission of the C-dots is clearly visible in aqueous suspensions even with the naked eye. The respective visible light for excitation was generated by a halogen lamp equipped with colored glass filters and a quartz-glass fiber. The visibility of the red emission is even better if the emitted light is observed through a red colored glass plate, which absorbs all light from excitation and emission except for red light (Figure 8b).

## 4. Discussion

On the one hand, heteroatom-containing C-dots have turned out most promising in regard to intense fluorescence, and on the other hand, to establishing emission features that are independent from the wavelength of excitation. In this regard, we present a simple one-pot, liquid-phase preparation of nitrogen-containing C-dots via a polyol-mediated synthesis. Accordingly, 1,2,4,5-tetracyanobenzene (TCB) was selected as a nitrogen-containing precursor. Polyethylene glycol 400 (PEG400) was selected as the solvent and stabilizing agent. The formation of the C-dots can be followed by the naked eye, when heating the TCB solution in PEG400 to 160 °C. At 100–120 °C the yellow solution turned to a deep black suspension of the C-dots. After 1 h of heating to 160 °C, the synthesis was finished. Transmission electron microscopy, X-ray diffraction, and infrared spectroscopy confirm the presence of crystalline C-dots with a diameter of 2–4 nm. Elemental analysis and thermogravimetry prove the incorporation of nitrogen and validate a nitrogen content of 24.4 wt. %. Suspensions of the C-dots are colloidally highly stable, even after dispersion in water.

The as-prepared, nitrogen-containing C-dots show intense fluorescence upon excitation at 400–500 nm and 600–700 nm. The emission comprises an excitation-wavelength-depending part at 400 to 650 nm as well as a strong emission peaking at 715 nm, which is independent from the wavelength of excitation. For the deep-red emission at 715 nm a remarkably high quantum yield of 69% was determined. The PEG400-stabilized nitrogen-containing C-dots can be dispersed in water and result in colloidally stable aqueous suspensions, which also show red emission. Taken together, the one-pot, liquid-phase synthesis of fluorescent nitrogen-containing C-dots can become interesting for reliable C-dot synthesis in general as well as in regard to deep-red emitting biomarkers for histology and imaging.

## References

[1] Panwar N., Soehartono A.M., Chan K.K., Zeng S., Xu G., Qu J., Coquet P., Yong K.-T., Chen X. (2019). Nanocarbons for biology and medicine: Sensing, imaging, and drug delivery. Chem. Rev..

[2] Hu C., Li M., Qiu J., Sun Y.-P. (2019). Design and fabrication of carbon dots for energy conversion and storage. Chem. Soc. Rev..

[3] Yuan F., Li S., Fan Z., Meng X., Fan L., Yang S. (2016). Shining carbon dots: Synthesis and biomedical and optoelectronic applications. Nano Today.

[4] Lim S.Y., Shen W., Gao Z. (2015). Carbon quantum dots and their applications. Chem. Soc. Rev..

[5] Hong G., Diao S., Antaris A.L., Dai H. (2015). Carbon nanomaterials for biological imaging and nanomedicinal therapy. Chem. Rev..

[6] Jiang K., Sun S., Zhang L., Lu Y., Wu A., Cai C., Lin H. (2015). Red, green, and blue luminescence by carbon dots: Full-color emission tuning and multicolor cellular imaging. Angew. Chem. Int. Ed..

[7] Scialabba C., Sciortino A., Messina F., Buscariono G., Cannas M., Roscigno G., Condorelli G., Cavallaro G., Giammona G., Mauro N. (2019). Highly homogeneous biotinylated carbon nanodots: Red-emitting nanoheaters as theranostic agents toward precision cancer medicine. ACS Appl. Mater. Interfaces.

[8] Huo F., Liang W., Tang Y., Zhang W., Liu X., Pei D., Wang H., Jia W., Jia P., Yang F. (2019). Full-color carbon dots with multiple red-emission tuning: On/off sensors, in vitro and in vivo multicolor bioimaging. J. Mater. Sci..

[9] Weissleder R. (2001). A clearer vision for in vivo imaging. Nat. Biotechnol..

[10] Ye R., Xiang C., Lin J., Peng Z., Huang K., Yan Z., Cook N.P., Samuel E.L.G., Hwang C.-C., Ruan G. (2013). Coal as an abundant source of graphene quantum dots. Nat. Commun..

[11] Tang Q., Zhu W., He B., Yang P. (2017). Rapid Conversion from carbohydrates to large-scale carbon quantum dots for all-weather solar cells. ACS Nano.

[12] Wang J., Wang C.F., Chen S. (2012). Amphiphilic egg-derived carbon dots: Rapid plasma fabrication, pyrolysis process, and multicolor printing patterns. Angew. Chem. Int. Ed..

[13] Sahu S., Behera B., Maiti T.K., Mohapatra S. (2012). Simple one-step synthesis of highly luminescent carbon dots from orange juice: Application as excellent bio-imaging agents. Chem. Commun..

[14] Peng H., Travas-Sejdic J. (2009). Simple Aqueous Solution Route to Luminescent Carbogenic Dots from Carbohydrates. Chem. Mater..

[15] Li L., Lu C., Li S., Liu S., Wang L., Cai W., Xu W., Yang X., Liu Y.G., Zhang R. (2017). A high-yield and versatile method for the synthesis of carbon dots for bioimaging applications. J. Mater. Chem. B.

[16] Dong H., Kuzmanoski A., Gößl D.M., Popescu R., Gerthsen D., Feldmann C. (2014). Polyol-mediated C-dots formation showing efficient Tb^3+^/Eu^3+^ emission. Chem. Commun..

[17] Liu Y., Xiao N., Gong N., Wang H., Shi X., Gu W., Ye L. (2014). One-step microwave-assisted polyol synthesis of green luminescent carbon dots as optical nanoprobes. Carbon.

[18] Dong H., Roming M., Feldmann C. (2015). Unexpected fluorescence of polyols and PEGylated nanoparticles derived from carbon dot formation. Part. Part. Syst. Charact..

[19] Li R.S., Liu J.H., Yang T., Gao P.F., Wang J., Liu H., Zhen S.J., Li Y.F., Huang C.Z. (2019). Carbon quantum dots-europium (III) energy transfer architecture embedded in electrospun nanofibrous membranes for fingerprint security and document counterspy. Anal. Chem..

[20] Hua X.-W., Bao Y.-W., Zeng J., Wu F.-G. (2019). Nucleolus-targeted red emissive carbon dots with polarity-sensitive and excitation-independent fluorescence emission: High-resolution cell imaging and in vivo tracking. ACS Appl. Mater. Interfaces.

[21] Shi X., Meng H., Sun Y., Qu L., Lin Y., Li Z., Du D. (2019). Far-red to near-infrared carbon dots: Preparation and applications in biotechnology. Small.

[22] Yang X., Cui F., Ren R., Sun J., Ji J., Pi F., Zhang Y., Sun X. (2019). Red-emissive carbon dots for “switch-on” dual function sensing platform rapid detection of ferric ions and L-cysteine in living cells. ACS Omega.

[23] Liu Y., Duan W., Song W., Liu J., Ren C., Wu J., Liu D., Chen H. (2017). Red emission B, N, S-co-doped carbon dots for colorimetric and fluorescent dual mode detection of Fe^3+^ ions in complex biological fluids and living cells. ACS Appl. Mater. Interfaces.

[24] Ding H., Yu S.-B., Wei J.-S., Xiong H.-M. (2016). Full-color light-emitting carbon dots with a surface-state-controlled luminescence mechanism. ACS Nano.

[25] Ge J., Jia Q., Liu W., Guo L., Liu Q., Lan M., Zhang H., Meng X., Wang P. (2015). Red-emissive carbon dots for fluorescent, photoacoustic, and thermal theranostics in living mice. Adv. Mater..

[26] De Mello J.C., Wittmann H.F., Friend R.H. (1997). An improved experimental determination of external photoluminescence quantum efficiency. Adv. Mater..

[27] Dong H., Chen Y.-C., Feldmann C. (2015). Polyol synthesis of nanoparticles: Status and options regarding metals, oxides, chalcogenides, and non-metal elements. Green Chem..

[28] Blasse G., Grabmaier C. (1994). Luminescent Materials.

[29] Iwata S., Tanaka J., Nagakura S. (1967). Absorption and emission spectra of 1, 2, 4, 5-tetracyanobenzene-naphthalene complex crystal. J. Am. Chem. Soc..

[30] Steudle W., Von Schuetz J.U., Moehwald H. (1978). Optical studies of the 1:1 CT crystal anthracene/TCNB: Mobile triplet excitons at 1.2 K. Chem. Phys. Lett..

[31] Levy D., Arnold B.R. (2005). Analysis of charge-transfer absorption and emission spectra on an absolute scale: Evaluation of free energies, matrix elements, and reorganization energies. J. Phys. Chem. A.

[32] Dong Y., Pang H., Yang H.B., Guo C., Shao J., Chie Y., Li C.M., Yu T. (2013). Carbon-based dots co-doped with nitrogen and sulfur for high quantum yield and excitation-independent emission. Angew. Chem. Int. Ed..

[33] Zhang Y.Q., Ma D.K., Zhuang Y., Zhang X., Chen W., Hong L.L., Yan Q.X., Yu K., Huang S.M. (2012). One-pot synthesis of N-doped carbon dots with tunable luminescence properties. J. Mater. Chem..

[34] Jaiswal A., Gosh S.S., Chattopadhyya A. (2012). One step synthesis of C-dots by microwave mediated caramelization of poly (ethylene glycol). Chem. Commun..

[35] Dong H., Kuzmanoski A., Wehner T., Müller-Buschbaum K., Feldmann C. (2017). Microwave-assisted polyol synthesis of water dispersible red-emitting Eu^3+^-modified carbon dots. Materials.

[36] Sun Y.P., Zhou B., Lin Y., Wang W., Shiral K.A., Fern O., Pathak P., Meziani M.J., Harruff B.A., Wang X. (2006). Quantum-Sized Carbon Dots for Bright and Colorful Photoluminescence. J. Am. Chem. Soc..

[37] Mondal S., Chatti M., Mallick A., Purkayastha P. (2014). pH triggered reversible photoinduced electron transfer to and from carbon nanoparticles. Chem. Commun..

